# Bacterial community profiles of soils from the Mount Makiling Forest Reserve, Philippines

**DOI:** 10.1128/mra.00936-26

**Published:** 2026-10-23

**Authors:** Armi R. Creencia, Eldrin D. L. R. Arguelles, Noel H. Tan Gana, Rose May Anne D. Capanzana, Jaimar P. Martinez, Jovenal C. Lanceras, Clavel Ayseri M. Salaan, Ralf Aeimos M. Del Castillo, Darlene Faye B. Cadao

**Affiliations:** 1 Philippine National Collection of Microorganisms (PNCM), National Institute of Molecular Biology and Biotechnology (BIOTECH), University of the Philippines Los Baños (UPLB) 54729, Los Baños, Laguna, Philippines; 2 UPLB Graduate School, Los Baños, Laguna, Philippines; Indiana University Bloomington, Bloomington, Indiana, USA

**Keywords:** microbial diversity, tropical forest, amplicon metagenomic analysis, soil microbiome

## Abstract

The Mount Makiling Forest Reserve harbors diverse microorganisms with potential biotechnological applications, yet bacterial taxonomic profiling across its watersheds remains limited. Amplicon metagenome sequencing targeting the 16S rRNA gene revealed Proteobacteria and Acidobacteriota as dominant phyla, with Actinobacteriota, Verrucomicrobiota, Chloroflexi, Myxococcota, Methylomirabilota, Bacteroidota, Nitrospirota, and Planctomycetota present at varying abundances.

## ANNOUNCEMENT

The Mount Makiling Forest Reserve (MMFR) is a protected tropical forest ecosystem characterized by variation in elevation, soil texture, and nutrient status. Its soils support diverse microbial communities involved in nutrient cycling, organic matter decomposition, and ecosystem stability (1, 2), and may harbor microorganisms, genes, and metabolites with potential applications in agriculture, biotechnology, and bioremediation (2, 3). Despite their ecological importance, Southeast Asian tropical forest microbiomes remain relatively understudied (4), and bacterial diversity across MMFR watersheds and environmental gradients has been poorly characterized (5–9).

Soil samples were collected from the Molawin-Dampalit, Cambantoc, Sipit, and Tigbi watersheds from September to November 2025. Fifteen sites spanning three elevation classes (<100, 100–200, and 200–300 masl) were sampled. At each site, three independent topsoil samples were collected from spatially separated points within a 5 × 5 m quadrat at 0–10 cm depth, yielding 45 samples (Table 1). Samples were transported at approximately 4°C and stored at −80°C until DNA extraction. Molawin and Dampalit were sampled separately for greater spatial representation but pooled for watershed-level comparisons.

**TABLE 1:** Sequence Read Archive accession number and library statistics through filtering, denoising, merging, and chimeric and non-bacterial sequence removal of V3–V4 region amplicons from the MMFR soils.

Sampling site	Sample code	Input	Filtered	Denoised	Merged	Input merged (%)	Non-chimeric	SRA accession no.
Cambantoc (<100 masl)	C1-1	66,376	64,127	51,378	25,020	37.69	23,395	SRX33587171
	C1-2	87,064	84,544	70,749	38,306	44	35,383	SRX33587138
	C1-3	72,757	70,842	56,285	27,176	37.35	25,684	SRX33587139
Cambantoc (100–200 masl)	C2-1	67,775	65,823	53,046	25,928	38.26	24,133	SRX33587140
	C2-2	74,199	71,817	55,513	25,267	34.05	23,763	SRX33587141
	C2-3	79,832	77,646	63,222	30,937	38.75	28,712	SRX33587142
Cambantoc (200–300 masl)	C3-1	73,935	71,863	58,341	28,507	38.56	26,796	SRX33587143
	C3-2	72,119	70,318	58,694	31,574	43.78	29,876	SRX33587144
	C3-3	78,233	75,936	62,593	32,926	42.09	31,260	SRX33587145
Molawin-Dampalit (<100 masl)	D1-1	187,607	32,722	26,575	10,343	5.51	9,766	SRX33587136
	D1-2	182,317	24,347	19,728	6,694	3.67	6,266	SRX33587137
	D1-3	186,489	32,778	27,037	10,558	5.66	9,964	SRX33587148
Molawin-Dampalit (100–200 masl)	D2-1	141,783	27,190	22,207	9,610	6.78	9,153	SRX33587159
	D2-2	166,072	27,757	23,170	9,672	5.82	9,346	SRX33587166
	D2-3	176,829	28,444	23,067	8,507	4.81	8,285	SRX33587167
Molawin-Dampalit (200–300 masl)	D3-1	172,337	28,921	23,702	8,826	5.12	8,570	SRX33587168
	D3-2	159,998	21,307	17,720	7,093	4.43	6,873	SRX33587169
	D3-3	149,079	22,754	18,055	6,333	4.25	6,080	SRX33587170
Molawin-Dampalit (<100 masl)	M1-1	175,567	23,300	18,440	6,761	3.85	6,353	SRX33586879
	M1-2	169,839	27,419	22,095	7,725	4.55	6,956	SRX33586880
	M1-3	199,763	35,569	29,056	11,199	5.61	10,522	SRX33586881
Molawin-Dampalit (100–200 masl)	M2-1	158,729	26,339	21,639	8,847	5.57	8,732	SRX33586882
	M2-2	200,618	28,532	23,665	9,541	4.76	9,256	SRX33586883
	M2-3	198,597	25,903	20,742	7,065	3.56	6,848	SRX33586884
Molawin-Dampalit (200–300 masl)	M3-1	154,106	23,747	19,357	7,584	4.92	7,030	SRX33586885
	M3-2	164,048	23,594	18,891	6,883	4.2	6,562	SRX33586886
	M3-3	172,798	28,370	23,251	8,239	4.77	8,006	SRX33586887
Sipit (<100 masl)	S1-1	108,230	104,154	87,903	46,054	42.55	41,536	SRX33587146
	S1-2	86,426	84,032	69,994	36,353	42.06	34,115	SRX33587147
	S1-3	91,812	89,301	71,332	32,741	35.66	29,666	SRX33587149
Sipit (100–200 masl)	S2-1	64,747	62,681	51,579	28,491	44	26,130	SRX33587150
	S2-2	79,269	77,093	62,433	31,095	39.23	28,649	SRX33587151
	S2-3	85,905	83,533	68,120	34,199	39.81	31,348	SRX33587152
Sipit (200–300 masl)	S3-1	67,025	64,865	55,179	30,751	45.88	26,531	SRX33587153
	S3-2	95,307	92,095	77,568	42,183	44.26	39,403	SRX33587154
	S3-3	65,443	63,673	53,156	29,847	45.61	27,885	SRX33587155
Tigbi (<100 masl)	T1-1	64,676	62,731	52,475	29,673	45.88	27,593	SRX33587156
	T1-2	67,524	65,265	52,876	27,009	40	25,473	SRX33587157
	T1-3	76,964	74,988	59,508	28,775	37.39	26,900	SRX33587158
Tigbi (100–200 masl)	T2-1	79,907	77,142	62,152	31,952	39.99	29,643	SRX33587160
	T2-2	75,880	73,855	59,953	31,439	41.43	29,045	SRX33587161
	T2-3	98,271	95,334	84,925	59,319	60.36	54,385	SRX33587162
Tigbi (200–300 masl)	T3-1	76,452	74,021	60,814	31,239	40.86	28,829	SRX33587163
	T3-2	86,479	83,898	68,794	35,522	41.08	32,460	SRX33587164
	T3-3	73,134	71,044	55,043	25,174	34.42	23,191	SRX33587165

Soil DNA was extracted using the E.Z.N.A. Soil DNA Kit (Omega Bio-tek, USA), assessed by 1% agarose gel electrophoresis, and quantified using a Shimadzu BioSpec-nano spectrophotometer. Libraries were prepared following the Illumina 16S Metagenomic Sequencing Library Preparation workflow (10). The V3–V4 region of the bacterial 16S rRNA gene was amplified using primers 341F (TCGTCGGCAGCGTCAGATGTGTATAAGAGACAGCCTACGGGNGGCWGCAG) and 805R (GTCTCGTGGGCTCGGAGATGTGTATAAGAGACAGGACTACHVGGGTATCTAATCC) with Illumina adapter overhangs. The 5-ng genomic DNA input was amplified with 5× reaction buffer, 1 mM of dNTP mix, 500 nM each of the universal primers, and Herculase II fusion DNA polymerase (Agilent Technologies, Santa Clara, CA, USA). PCR cycling consisted of 95°C for 3 min; 25 cycles of 95°C for 30 s, 55°C for 30 s, and 72°C for 30 s; followed by 72°C for 5 min. Amplicons were purified with AMPure beads (Agencourt Bioscience, Beverly, MA, USA), subjected to a second 10-cycle PCR for library construction, purified again, quantified with PicoGreen, assessed using TapeStation D1000 ScreenTape (Agilent Technologies, Waldbronn, Germany), normalized, pooled, and quantified by qPCR. Sequencing was performed on an Illumina MiSeq platform using 2 × 300-bp paired-end reads (Illumina, San Diego, CA, USA).

Demultiplexed reads were processed in QIIME 2 v2024.10 using DADA2 for denoising, merging, chimera removal, and ASV inference (11). Reads were trimmed to 15 bp and truncated to 275 bp forward and 225 bp reverse, yielding 956,352 high-quality non-chimeric reads. Taxonomy was assigned using the SILVA v138.1 classifier (12, 13), and non-bacterial and unassigned sequences were removed. Visualization was performed in R v4.3.3 (14).

**Fig 1:**
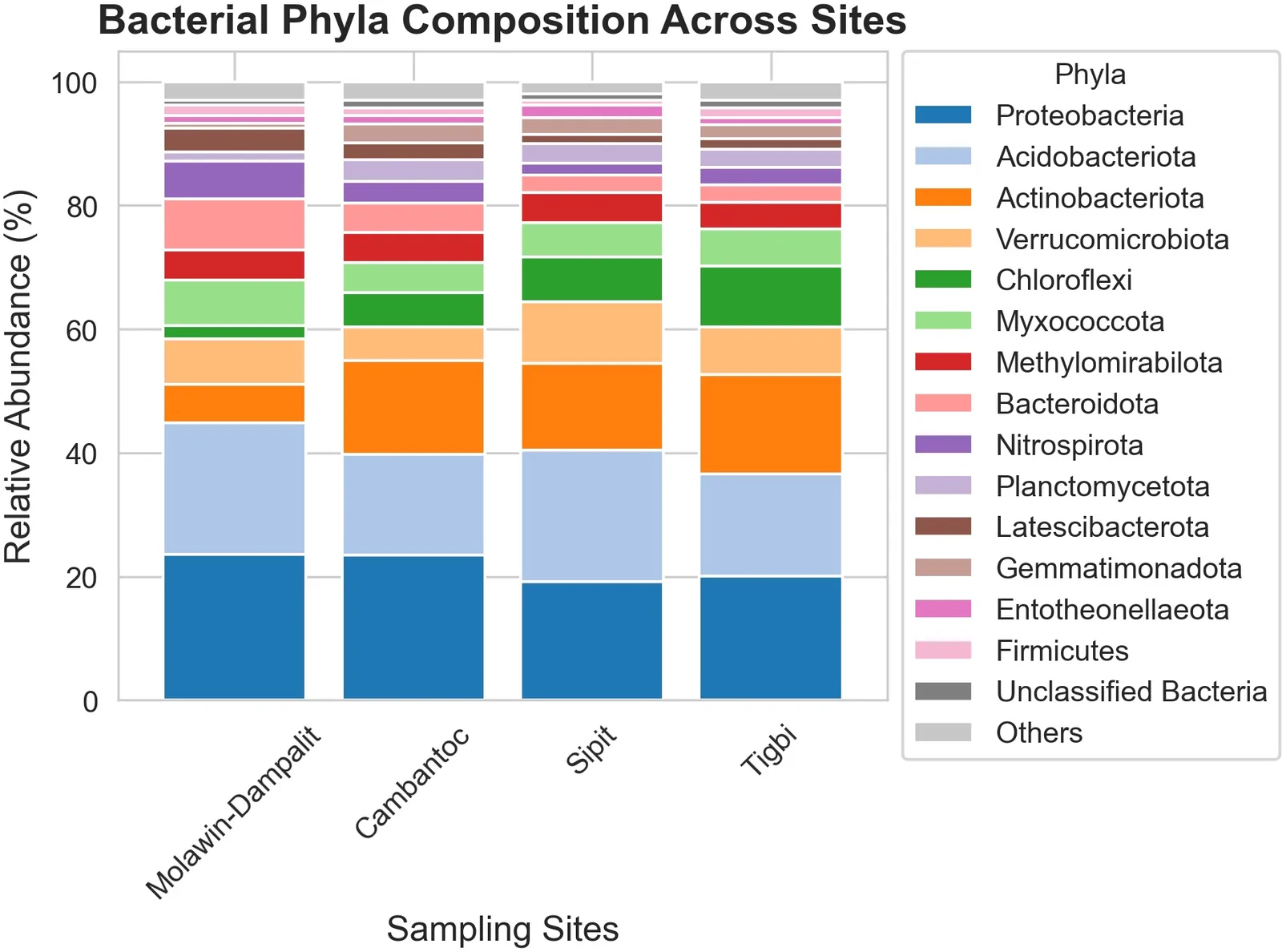
Phylum-level bacterial community profiles of the Mount Makiling forest soils as determined by amplicon metagenome sequencing of the V3–V4 region of the 16S rRNA gene. Each bar represents the combined data from the soil samples for each site. Each colored stacked box represents a bacterial taxon.

Proteobacteria (21.57%), Acidobacteriota (18.85%), and Actinobacteriota (12.85%) were the dominant phyla across sites (Fig. 1). Differences in relative abundance among watersheds likely reflect variation in local soil and environmental conditions. These findings provide baseline data on Philippine tropical forest soil microbiomes and highlight MMFR as a potential source of valuable microbial resources.

## Data Availability

The raw sequences obtained in this study are available in the National Center for Biotechnology Information (NCBI) Sequence Read Archive (SRA) under BioProject number PRJNA1469809.
